# Splenic artery aneurysm rupture post-anterior cervical discectomy and fusion: Case report & literature review

**DOI:** 10.1016/j.ijscr.2022.107704

**Published:** 2022-09-27

**Authors:** Moaiad Mohammed Hussein, Mustafa Al-Mollah, Tariq Kanaan

**Affiliations:** Division of Neurosurgery, Department of Special Surgery, University of Jordan, Amman, Jordan

**Keywords:** ACDF, Aneurysm rupture, Splenectomy, Anterior cervical spinal surgery

## Abstract

**Introduction and importance:**

Anterior cervical discectomy and fusion (ACDF) is a regular surgical procedure for correcting spinal deformities and pain relief. There are several rare complications of ACDF, one of which is postoperative hematomas. Here, we report an unexpected case of intra-abdominal hematoma after ACDF with no prior abdominal symptoms or underlying conditions identified since admission. This report will describe the events and interventions that took place for this patient.

**Case description:**

The patient is a 44-year-old female with a history of neck pain of four-month duration. On Magnetic Resonance Imaging (MRI), a degenerative cervical disk (C5-C6) was identified. Prior surgical history is significant for a C4-C5 ACDF 3 years ago. An anterior cervical discectomy and fusion was performed and the patient was doing well relatively post-surgery. However, in less than 24 h, the patient complained of severe abdominal pain. An abdominal Computerized topography angiogram (CTA) scan revealed internal bleeding and a splenic aneurysm rupture. The patient immediately underwent an urgent laparotomy and splenectomy.

**Clinical discussion:**

Splenic artery aneurysm incidence is rare and is detected incidentally by imaging technology in asymptomatic patients or upon rupture. Splenic artery aneurysm rupture can be spontaneous and unpredictable in previously undiagnosed patients leading to life-threatening symptoms of intra-abdominal hemorrhage.

**Conclusion:**

Patients undergoing ACDF should be monitored closely following surgery for any complications. Physicians should consider the possibility of any signs of hematoma due to underlying conditions that are undiagnosed in order to treat accordingly.

## Highlights

• Unexpected case of intra-abdominal hematoma post-anterior cervical discectomy and fusion (ACDF) due to splenic artery aneurysm.
• True splenic artery aneurysms (SAA) are rare but potentially fatal, they are the third most common abdominal aneurysms.
• As much as 80% of cases of splenic artery aneurysms are asymptomatic.
• Surgical patients should be evaluated prior to operations and post-operative monitoring should be performed to prevent/treat complications.

## 1 Introduction

Anterior cervical discectomy and fusion (ACDF) is an operation performed to remove a deformed or herniated disc in the neck. An incision is made in the throat to approach the disc and remove it. A bone graft or a cage is then inserted to prevent the related vertebral bones from collapsing [1]. Despite this surgery having a high success rate, it has multiple possible complications. Although uncommon, complications such as dysphagia and postoperative hematomas have been reported which can ultimately prove fatal if untreated. On very rare occasions, these complications may require secondary surgery. The discovery of any pre-disposing factors to these complications should warrant proactive measures to prevent these complications from taking place and to improve the overall outcome of the surgical procedure [2]. This case report has been written to be in line with the SCARE criteria [3].

## 2 Case presentation

### 2.1 Patient information

A 44-year-old female presented to our institution (Jordan University Hospital Outpatient Clinics) with neck pain of four-month duration. The patient stated that her pain was associated with right brachialgia (pain score 2/10) at C5 as well as numbness and paresthesia at C6. Previous surgical history includes a C4/C5 ACDF with cage size 4 in 2017. The patient is a 7-pack year smoker. The patient was not on any medications. Also, the patient has no history of hypertension, atherosclerosis, Marfan disease, Ehlers-Danlos syndrome, or vasculitis. However, the patient's family history is significant for her mother who has been diagnosed with hypertension.

### 2.2 Clinical findings

On physical examination, the patient demonstrated that she suffered from a spastic gait at both lower limbs. In addition, bilateral Hoffman and Babinski signs were seen. An MRI showed C5-C6 Prolapsed Intervertebral Disc (PID) which prompted an urgent ACDF. It should be noted that the rest of the physical examination including vitals were all within normal limits.

### 2.3 Follow-up and outcomes

**Fig. 1:**
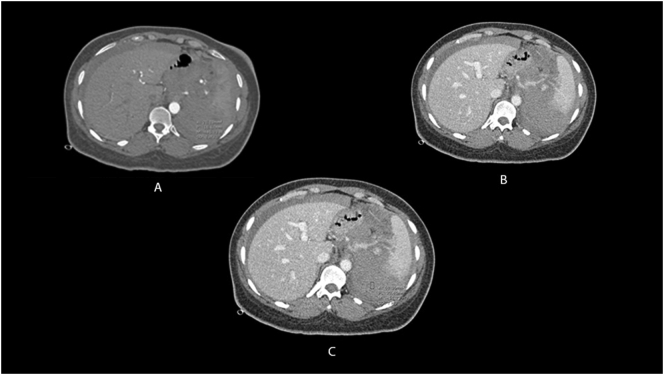
Abdominal CT angiogram arterial phase (a), venous phase (b&c).

**Fig. 2:**
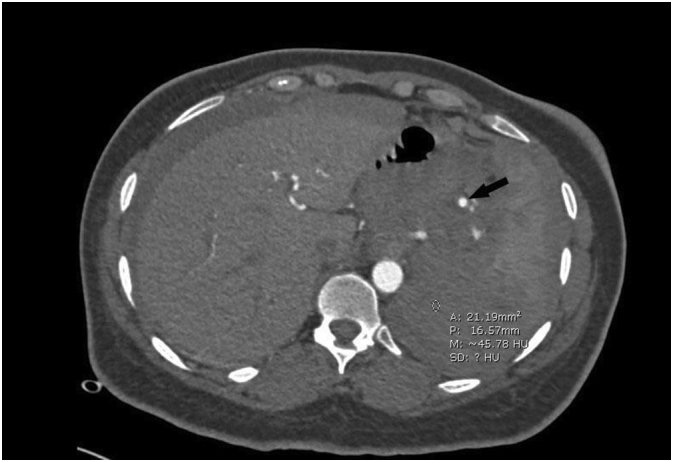
Shows Abdominal CT angiogram arterial phase, the arrow points at the site of the aneurysm.

**Fig. 3:**
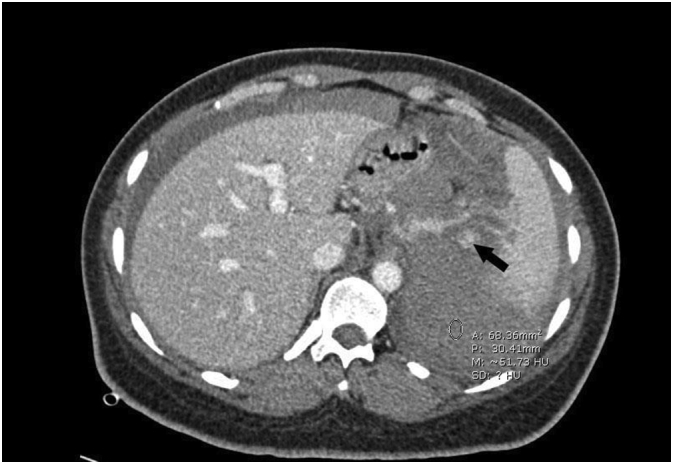
Shows abdominal CT angiogram venous phase with an arrow pointing at the site of the aneurysm and a circle indicating extensive hemoperitoneum (50 HU), no active contrast extravasation could be seen.

**Fig. 4:**
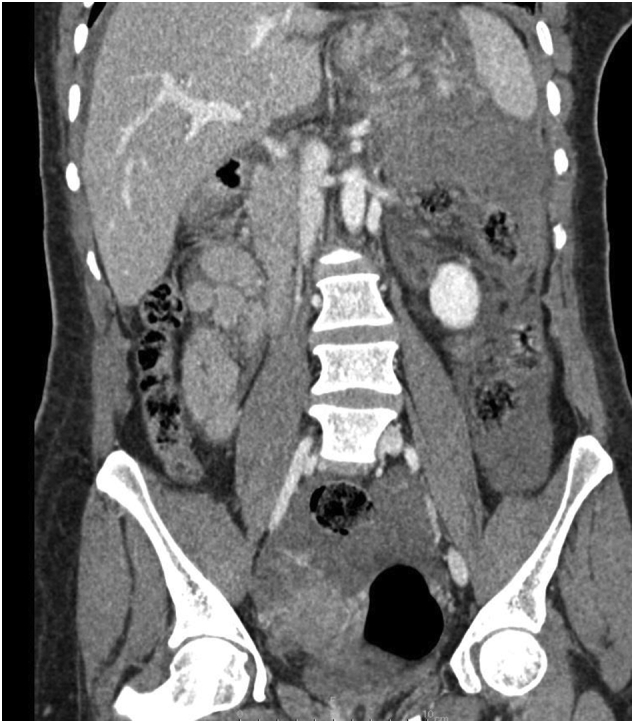
Shows a coronal reconstruction CT scan in the venous phase showing extensive hemoperitoneum.

The patient was admitted for surgery and successfully underwent her ACDF procedure done by Dr. Tariq Kanaan. However, it was complicated by sudden severe hypertension of over 200/100 mmhg, followed by multiple episodes of hypotension. Initially within the first four hours post-op, the patient was doing relatively well and had stable vitals. However, the patient suddenly began to experience generalized abdominal pain. This continuous pain was described has heavy and dull and did not radiate anywhere else. The patient stated her pain was not associated with any nausea or vomiting. Blood tests revealed the patient had a troponin level of 1050 ng/L while Electrocardiography (ECG) showed normal sinus rhythm. A cardiology consult recommended serial troponin measurements, however the patient became hypotensive with a systolic blood pressure of 75 mmHg and a diastolic blood pressure of 50 mmHg. The patient's pulse was 80 bpm. Concurrently, hemoglobin levels were reported to be 6.5 g/dL. An abdominal CTA scan was preformed immediately which revealed internal hemorrhaging and a splenic aneurysm rupture (Fig. 1, Fig. 2, Fig. 3, Fig. 4). The patient underwent an urgent laparotomy and splenectomy by Dr. Mohammad El Muhtaseb and an abdominal drain was inserted. An emergent exploratory laparotomy was warranted over endovascular coil embolization due to the patient's condition being in hypovolemic shock.

Intra-operation findings during exploratory laparotomy and splenectomy revealed bleeding from the splenic artery, multiple liquified clots (approximately one liter) in addition to pelvic, retro-gastric and splenic flexure hematomas. Evacuation of hematomas and clots was preformed, and ligation of the splenic artery was done. After stabilization, no active bleeding was seen. Subsequently, post-operation the patient was extubated and sent to the intensive care unit with an abdominal drain, foleys catheter and a left radial arterial line.

The macroscopic histopathological examination of the resected specimen from the splenectomy included a spleen measuring 10 × 6.5 × 4 cm with an attached fragment of the hilum measuring 8 × 6 × 3.5 cm and a fragment of the omentum measuring 8 × 6 × 3 cm. The spleen was weighed at 175.7 g. Step sectioning of the omentum revealed black cut surface with multiple yellow foci while step sectioning of the hematoma showed a hemorrhagic black cut surface. The splenic vessels could not be identified with certainty because of the hematoma. Step sectioning of the spleen was unremarkable. Microscopic histopathological examination of splenic tissue sections showed congestion along with fragments of fat necrosis and hematoma. Sections taken from the hematoma revealed splenic artery with blood dissecting through the media, indicating the presence of wall rupture.

The histopathological diagnosis concluded that the resected specimen showed congested splenic tissue with hematoma and that the specimen was negative for malignancy.

Forty-eight hours following surgery, hemoglobin and complete blood count levels began to normalize.

## 3 Discussion

An aneurysm is an outward protrusion that is balloon-like at a weak point in an artery, thus the artery would have an increase in its focal diameter by at least 50 % or more than its expected diameter [4]. True splenic artery aneurysms (SAA) are rare but potentially fatal, they are the third most common abdominal aneurysms after those of the Aorta and the common Iliac artery [5]. True splenic artery aneurysms make up to 60 % of cases of splenic artery aneurysms as these may include the presentation of pseudoaneurysms [6]. The incidence of true splenic artery aneurysms in women is four folds that of men [5]. In addition, 5 % of intra-abdominal aneurysms are SAAs and they are the most common visceral aneurysms [6]. According to the most recently released statistics, the incidence of SAA is in 0.01 % to 0.2 % of patients [7].

Splenic artery aneurysms were first described in 1770 by Beaussier, however, minimally invasive surgical techniques were not modalities of treatment until 1993 [8]. This is when laparoscopy was first used for the resection of splenic artery aneurysms [8]. Fortunately, concurrent advancements in imaging technology led to an increase in the rate of incidental detection of visceral aneurysms [8].

Most true SAAs appear in the main body of the splenic artery. About 74–87 % arise in the distal third, 20–22 % in the middle third, and less than 6 % in the proximal third [6]. Etiology of SAA formation remains relatively idiopathic, however there are major risk factors for their development including portal hypertension, atherosclerosis, smoking, collagen disease and pregnancy [6].

As much as 80 % of cases of SAA are asymptomatic [5]. However, when they are symptomatic, the patient may present with left epigastric pain or a hypotensive shock secondary to rupture which can be critical [9]. Symptomatic cases often involve enlargement of the aneurysm by more than 2 cm and they account for 20 % of all SAA cases with spontaneous rupture being the most common complication in those patients [10]. Some (2–10 %) patients will present spontaneous rupture [5], with mortality of 10–40 %. Rupture can be instant or can happen in two stages, which occur in 20 to 25 % of cases [6]. Two-staged rupture occurs after the blood clot clogs Winslow's foramen then the clot would rupture intraperitoneally, resulting in abdominal pain and distention. Another possibility would be a case of double-rupture phenomenon, in which the SAA would rupture causing hemodynamic instability which is relatively conciliated as the hemorrhage is held within the lesser omental sac. But if the hemorrhage is sustained for a prolonged period, the lesser sac can rupture into the peritoneal cavity with recurring hemodynamic instability [11].

Diagnosis of SAA is done by suspicion of SAA by imaging techniques such as ultrasound of the abdomen, abdominal computed tomography and magnetic resonance imaging. Despite that, contrast CT is the imaging technique of choice to check for SAA [6]. However most SAAs are normally detected incidentally in asymptomatic patients or in a minority of symptomatic cases upon rupture.

Interventional treatments include open surgical, laparoscopic, percutaneous abdominal and endovascular repair, laparoscopic repair of AAE is the most commonly used intervention of all aneurysms, due to the position of the SAA and straightforward approach [6].

In conclusion, patients undergoing major surgeries should be monitored closely following surgery for any complications perhaps unrelated to the procedure itself. Physicians should consider the wide variety of possibilities of post-op complications that might be due to underlying undiagnosed conditions.

## Consent for publication

Written informed consent was obtained from the patient for publication of this case report and accompanying images. A copy of the written consent is available for review by the Editor-in Chief of this journal on request.

## Sources of funding

This study did not receive any funding support.

## Ethical approval

Ethical approval was obtained from the Institutional Review Board of Jordan University Hospital.

## Guarantor

Dr. Tariq Kanaan

## Research registration number

1. Name of the registry: Research registry
2. Unique identifying number or registration ID: researchregistry8257
3. Hyperlink to your specific registration (must be publicly accessible and will be checked):

https://www.researchregistry.com/browse-the-registry#home/registrationdetails/6310a4e443cca30022666985/

## Provenance and peer review

Not commissioned, externally peer-reviewed.

## CRediT authorship contribution statement

**Moaiad Hussein Hussein:** Conceptualization, Writing – original draft, Investigation. **Mustafa Al-Mollah:** Investigation, Writing – review & editing, Resources. **Tariq Kanaan:** Supervision, Writing – review & editing.

## Declaration of competing interest

There is no conflict of interest to be declared.
